# Outcomes of Distal Third Femur Fractures in Patients 18 Years and Older: A Pilot Study

**DOI:** 10.7759/cureus.55136

**Published:** 2024-02-28

**Authors:** Gregory R Roytman, Sahir S Jabbouri, Jamieson O'Marr, Akshay Raghuram, Brian Beitler, Suhail Irshad, Brianna R Fram, Brad J Yoo, Michael P Leslie, Matthew D Riedel, Steven M Tommasini, Daniel H Wiznia

**Affiliations:** 1 Orthopaedics and Rehabilitation, Yale School of Medicine, New Haven, USA; 2 Orthopaedics, Mount Sinai Hospital, New Haven, USA

**Keywords:** outcome assessment, demography, cluster analysis, dual plate construct, retrograde intramedullary femoral nail, lateral locking plate, distal femur fractures

## Abstract

Introduction: The selection of the most optimal fixation method for fractures of the distal femur, whether intramedullary nail (NL), lateral locking plate (PL), or nail/plate (NP) is not always clear. This study retrospectively evaluates surgical patients with distal femur fractures and introduces a pilot study using cluster analysis to identify the most optimal fracture fixation method for a given fracture type.

Methods: This is a retrospective cohort study of patients 18 years and older with an isolated distal femur fracture who presented to our Level-1 trauma center between January 1, 2012, and December 31, 2022, and obtained NL, PL, or NP implants. Patients with polytrauma and those without at least six months of follow-up were excluded. A chart review was used to obtain demographics, fracture classification, fixation method, and postoperative complications. A cluster analysis was performed. The following factors were used to determine a successful outcome: ambulatory status pre-injury and 6-12 months postoperatively, infection, non-union, mortality, and implant failure.

Results: A total of 169 patients met inclusion criteria. No statistically significant association between the fracture classification and fixation type with overall outcome was found. However, patients treated with an NP (n = 14) had a success rate of 92.9% vs only a 68.1% success rate in those treated with a PL (n = 116) (p = 0.106). The most notable findings in the cluster analysis (15 total clusters) included transverse extraarticular fractures demonstrating 100% success if treated with NP (n = 6), 50% success with NL (n=2), and 78.57% success with PL fixation (n=14). NP constructs in complete articular fractures demonstrated success in 100% of patients (n = 5), whereas 77.78% of patients treated with NL (n = 9) and 61.36% of those treated with PL (n = 44).

Conclusions: Plate fixation was the predominant fixation method used for distal third femur fractures regardless of fracture classification. However, NP constructs trended towards improved success rates, especially in complete intraarticular and transverse extraarticular fractures, suggesting the potential benefit of additional fixation with these fractures. Cluster analysis provided a heuristic way of creating patient profiles in patients with distal third femur fractures. However, a larger cohort study is needed to corroborate these findings to ultimately develop a clinical decision-making tool that also accounts for patient specific characteristics.

## Introduction

Distal femur fractures represent approximately 3% to 6% of all femur fractures [1]. In the United States, the incidence of distal femur fractures is approximately 37 per 100,000 person-years [2]. These fractures are typically seen after a low-energy mechanism (e.g., simple fall) in elderly women or a high-energy mechanism (e.g. motor vehicle crash) in young adults [2].

Various fixation constructs are available for the management of distal femur fractures, including the use of a retrograde intramedullary nail (NL), anatomic locking plate (PL), or a nail/plate (NP) dual construct. Treatment with an NL construct results in minimal biological soft tissue disruption and preservation of the blood supply at the fracture site, which optimizes fracture healing [3]. In addition, an NL construct typically allows immediate full weight bearing postoperatively [4]. NL constructs have traditionally been used in extraarticular distal femur fractures (33A) as well as some partial articular (33B) fractures. However, they can also be used for certain complete intraarticular (33C) fractures if combined with periarticular screws to achieve additional fixation [5].

PL fixation is typically used for periarticular (33B and 33C) fractures when NL fixation is difficult to achieve due to bone quality, fracture comminution, and/or extent of the intraarticular involvement [6]. It can also be used as a bridging construct for more comminuted extraarticular distal femur fractures (33A3.2 and 33A3.3). Patients typically are non-weight bearing or partial weight bearing postoperatively for intraarticular fracture patterns but some patients with extraarticular fracture patterns are allowed early weightbearing as tolerated [7].

Hoskin et al. suggested that an NL construct may be superior to anatomic PL for fixation of distal femur fractures as evidenced by higher reported quality-of-life at six months postoperatively and improved radiographic findings up to one year postoperatively [8]. In a systematic review, Jankowski et al. found a more than 90% union rate for distal femur fractures treated with either NL or PL and suggest that both constructs are acceptable methods of treatment for native distal femur fractures [9].

More recently, there has been an increase in the use of NP combination constructs for distal femur fractures, especially for patients at higher risk of nonunion (i.e., those with open, comminuted metaphysis fractures), patients with osteopenic bone and/or patients who are noncompliant [10]. An NP construct can allow for an additional load-sharing construct that can enhance fixation as well as expedite full weight bearing and fracture healing [11].

Ultimately, the choice of implant is typically determined by fracture type, bone quality, and surgeon experience. Given the complexity of distal femur fractures and the multiple treatment options available, there is a lack of clear consensus for the management of these fractures. A more systematic, data-driven approach to the management of distal femur fractures is needed. This 10-year retrospective study not only explores clinical outcomes of distal femur fractures managed with either an NL, PL, or NP dual construct but also, through a pilot cluster analysis, uniquely implements a preliminary outcomes-based surgical decision-making tool with the potential to assist surgeons with implant selection in the future.

## Materials and methods

The study protocol was submitted to Yale University’s Institutional Review Board (IRB) and an exemption under 45 CFR 46.104(d)(4) was obtained. This was a retrospective cohort study of patients 18 years and older with an isolated distal femur fracture who presented to our institution’s Level-1 trauma centers between January 1, 2012, and December 31, 2022. Current Procedural Terminology ® (CPT) codes 27511, 27513, and 27514 were used to identify the study group. Patients must have received treatment with an NL, PL, or NP to be included in the study. Patients with polytrauma or additional injuries that could impact postoperative recovery/ambulation were excluded. Periprosthetic fractures and patients who did not have at least six months of follow-up were also excluded. Patients with pathological fractures such as those resulting from carcinoma, were also excluded.

**Figure 1:**
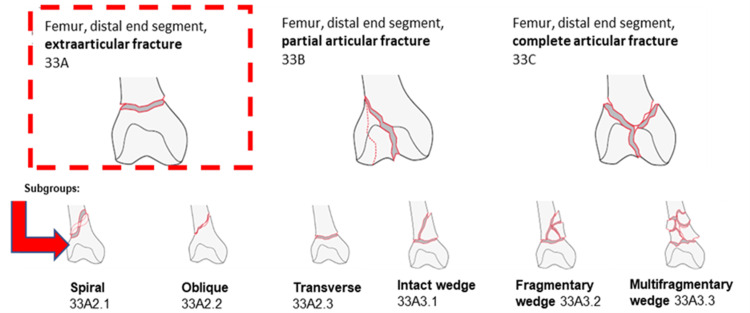
AO Fracture Classification System for distal femur fractures. (Copyright by AO Foundation, Switzerland) The image has a copyright by AO Foundation, Switzerland, and the authors have obtained permission to use this graphic.

A chart review was performed and patient age, sex, race, body mass index (BMI), Charlson Comorbidity Index (CCI), smoking history, and date of injury were documented. Preoperative X-ray and CT scans (when available) were used to appropriately classify the distal femur fracture based on the AO Distal Femur Fracture Classification system (Figure 1) [12].

Method of fixation (NL, PL, or NP) were documented. Complications, including implant failure, infection, need for reoperation, and mortality were documented. Postoperative radiographs and attending progress notes describing patient symptoms were used at 6-12 months to identify fracture union, nonunion, and malunion. Although the standard for assessment of fracture union is typically at nine months [13], we have chosen to extend the period that we consider follow-ups because surgeons may see patients for follow-up earlier or later than nine months for a variety of practical reasons. To capture those data, we have therefore extended the time range to 6-12 months for this study. 

Pre-operative and 6-12-month postoperative ambulatory status was noted for each patient, as determined by four pre-defined categories with respectively assigned numerical values: walking without assistance (0), requiring cane (1), requiring walker (2), wheelchair-bound (3), or bed-bound (4). The ambulatory status numerical values of each patient at 6-12 months follow-up were subtracted from the pre-operative ambulatory status numerical values to identify a change in ambulatory status (Table 1). A value of 0 indicated a return to baseline function, a negative value indicated worse ambulatory function and a positive value indicated improved function postoperatively compared with pre-injury.

**Table 1:** Calculation of change in ambulatory status from just prior to surgery (pre-injury) vs. 6-12 months follow-up after surgery (post-op). Change in Ambulatory Status = (Pre-injury) – (6-12 months follow-up)

Change in Ambulatory Status	
Pre-injury vs 6-12 months follow-up	
Type of Ambulation	Numerical Assignment
Walking without assistance	0
Requires cane	+1
Requires walker	+2
Wheelchair bound	+3
Bedbound	+4

The outcomes were binary: either successful or non-successful surgery. Non-successful surgery was defined as a patient having an infection, mortality, non-union of fracture, implant failure, or change in an ambulatory status less than a “-1” as determined by chart review. The presence of any one of these events constituted a non-successful surgery. Successful surgery was defined as a surgery in which the previously mentioned events were absent based on a chart review of follow-up at 6-12 months after the operation.

Clinical characteristics

A univariate analysis was performed to examine the relationship between fracture classification and both fixation type and clinical outcome using the Fisher’s Exact Test. Fisher's Exact Test was also used to evaluate for a relationship between fixation type and clinical outcome.

Cluster analysis

Cluster analysis was used as it allows the grouping of patients based on pertinent clinical characteristics to identify which characteristics lead to a certain outcome [14]. A partitioning-around medoids (PAM) algorithm was used to calculate Gower distances between patients. Gower distance is an alternative to the purely numerical Euclidean distance which allows for consideration of both numerical and categorical data. A Silhouette Analysis allowed us to determine the desired number of clusters. Our criteria for an appropriate number of clusters would be one that would be sufficiently high (above 0.7), [14] yet also allow for unique representation of two variables: AO Distal Femur Fracture Classification and fixation type (NL, PL, or NP). These variables were the characteristics that were deemed to be most clinically relevant. By minimizing variables, these two clinical characteristics were used in the model to predict surgical outcomes. The requisite number of clusters was therefore defined by proximity in Gower distance using the clinical characteristics.

## Results

Clinical characteristics

After final exclusions and extraction, 169 patients with distal femur fractures were included in our analysis. The patients had an average age of 66.1 years (SD 17.4) with an average BMI of 31.3 (SD 8.8) and were majority female (68.6%) (Table 2).

**Table 2:** Demographic characteristics of the distal femur fracture cohort.

Demographic	Number (%) (based on n = 169)
Age: mean ± SD	66.1 ± 17.4
Sex: n (%)	
Male	53 (31.4)
Female	116 (68.6)
Race: n (%)	
Black	22 (13.0)
White	132 (78.1)
Hispanic/Latino	11 (6.5)
Asian	1 (0.6)
Other	3 (1.8)
BMI: mean ± SD	31.3 ± 8.8
Smoking Status	
Yes	51 (30.2)
No	118 (69.8)
Charleson Comorbidity Index (mean ± SD)	4.4 ± 4.1

The most common fracture classifications were complete articular 33C (34.3%), oblique extraarticular 33A2.2 (18.9%), and transverse extraarticular 33A2.3 (12.4%) (Table 3). Of the total, 68.6% of constructs were PL, followed by 23.1% NL, and 8.3% NP. Overall, 72.2% of surgeries were considered to have a successful outcome based on our criteria (Table 3).

**Table 3:** The percentage of each AO distal femur fracture classification The percentage of each AO distal femur fracture classification, fixation type, and outcome – the main clinical characteristics assessed for in our cohort.

	Number (%) (based on n = 169)
Fracture Classification	
33A2.1	17 (10.1)
33A2.2	32 (18.9)
33A2.3	21 (12.4)
33A3.1	3 (1.8)
33A3.2	5 (3.0)
33A3.3	15 (8.9)
33B	18 (10.7)
33C	58 (34.3)
Fixation Type	
Plate	116 (68.6)
Nail	39 (23.1)
Nail and Plate	14 (8.3)
Outcome	
Success	122 (72.2)
Not Successful	47 (27.8)

On univariate analysis, fracture classification was significantly associated with fixation type with plate fixation being more common for extraarticular 33A (59.1%), partial articular 33B (94.4%), and complete articular 33C (75.9%) fractures (Table 4, p = 0.022). No statistically significant associations between the fracture classification and outcome (Table 5, p = 0.414) and fixation type with outcome (Table 6, p = 0.1058) were found. However, patients treated with an NP (n = 14) had a success rate of 92.9% vs only a 68.1% success rate in those treated with a PL (n = 116) (Table 6).

**Table 4:** Fisher exact test assessing association between AO distal femur fracture classification and fixation type.

	AO Distal Femur Fracture Classification			
	33A (n=93)	33B (n=18)	33C (n=58)	Fisher exact test p-value
Fixation Type n (%)				0.022
Plate	55 (59.1)	17 (94.4)	44 (75.9)	
Nail	29 (31.2)	1 (5.6)	9 (15.6)	
Nail and Plate	9 (9.7)	0 (0)	5 (8.6)	

**Table 5:** Fisher exact test assessing association between AO distal femur fracture classification and outcome.

	AO Distal Femur Fracture Classification			
	33A (n=93)	33B (n=18)	33C (n=58)	Fisher exact test p-value
Outcome n (%)				0.414
Successful	68 (73.1)	15 (83.3)	39 (67.2)	
Not Successful	25 (26.9)	3 (16.7)	19 (32.8)	

**Table 6:** Fisher exact test assessing the association between fixation type and outcome.

	Fixation Type			
	Plate (n=116)	Nail (n=39)	Nail and Plate (n=14)	Fisher exact test p-value
Outcome n (%)				0.1058
Successful	79 (68.1)	30 (76.9)	13 (92.9)	
Not Successful	37 (31.9)	9 (23.1)	1(7.1)	

About 27.8% of patients were deemed to have a non-successful surgery with a two-point drop in ambulatory status (n = 18) and nonunion (n = 12) as being the most common reasons (Table 7). Notably, complete intraarticular 33C fractures experienced the most post-operative ambulatory-related reason for non-successful surgery. Of those with complete intraarticular fracture, 26.32% had non-union (n = 5), 26.32% had a 2-point drop in ambulatory status (n = 5), 21.05% had implant failure (n = 4), and 10.53% experienced mortality (Table 8).

**Table 7:** Reasons for non-successful surgeries with percentages given out of total non-successful surgeries.

Reason for Non-Successful Surgery	n (%)
Two-Point Drop in Ambulatory Status Score	18 (38.30%)
Non-Union	12 (25.53%)
Mortality	5 (10.64%)
Implant Failure	4 (8.51%)
Three-Point Drop in Ambulatory Status Score	3 (6.38%)
Infection	2 (4.26%)
Greater than 1 Reason	3 (6.38%)
TOTAL	47

**Table 8:** The reasons for non-successful surgeries shown by fracture classification. Ambulatory status drop is given by the ambulatory status change based on the score in Table 1. Greater than 1 reason captures more than one of the previously mentioned reasons.

Fracture Classification								
	2-point ambulatory status drop	3-point ambulatory status drop	Implant Failure	Infection	Mortality	Greater than 1 Reason	Non-union	TOTAL
33A2.1	1 (100.00%)	0 (0.00%)	0 (0.00%)	0 (0.00%)	0 (0.00%)	0 (0.00%)	0 (0.00%)	1
33A2.2	4 (44.44%)	1 (11.11%)	0 (0.00%)	0 (0.00%)	2 (22.22%)	0 (0.00%)	2 (22.22%)	9
33A2.3	3 (75.00%)	0 (0.00%)	0 (0.00%)	0 (0.00%)	0 (0.00%)	1 (25.00%)	0 (0.00%)	4
33A3.1	0 (0.00%)	1 (50.00%)	0 (0.00%)	0 (0.00%)	0 (0.00%)	0 (0.00%)	1 (50.00%)	2
33A3.2	0 (0.00%)	0 (0.00%)	0 (0.00%)	1 (100.00%)	0 (0.00%)	0 (0.00%)	0 (0.00%)	1
33A3.3	4 (50.00%)	0 (0.00%)	0 (0.00%)	0 (0.00%)	0 (0.00%)	1 (12.50%)	3 (37.50%)	8
33B	1 (33.33%)	0 (0.00%)	0 (0.00%)	0 (0.00%)	1 (33.33%)	0 (0.00%)	1 (33.33%)	3
33C	5 (26.32%)	1 (5.26%)	4 (21.05%)	1 (5.26%)	2 (10.53%)	1 (5.26%)	5 (26.32%)	19

Of the patients who had a non-successful surgery and were treated with a PL, 40.54% had unsuccessful surgeries because of a 2-point drop in ambulatory status (n = 15) and 29.73% had unsuccessful surgeries because of non-union (n = 11). Of the patients who were deemed to have a non-successful surgery and were treated with an NL, reasons for non-successful surgery were evenly split (22.22%, n = 2) between the 2-point drop in ambulatory status, a 3-point drop in ambulatory status, and mortality. Only one patient experienced a non-successful surgery when treated with an NP with the reason being a 2-point drop in ambulatory status (Table 9).

**Table 9:** The reasons for non-successful surgeries shown by fixation type. Ambulatory status drop is given by the ambulatory status change based on the score in Table 1. Greater than 1 reason captures more than one of the previously mentioned reasons.

Fixation Type								
	2-point ambulatory status drop	3-point ambulatory status drop	Implant Failure	Infection	Mortality	Greater than 1 Reason	Non-union	TOTAL
Plate	15 (40.54%)	1 (2.70%)	4 (10.81%)	1 (2.70%)	3 (8.11%)	2 (5.41%)	11 (29.73%)	37
Nail	2 (22.22%)	2 (22.22%)	0 (0.00%)	1 (11.11%)	2 (22.22%)	1 (11.11%)	1 (11.11%)	9
Nail and Plate	1 (100.00%)	0 (0.00%)	0 (0.00%)	0 (0.00%)	0 (0.00%)	0 (0.00%)	0 (0.00%)	1

Cluster analysis

**Figure 2:**
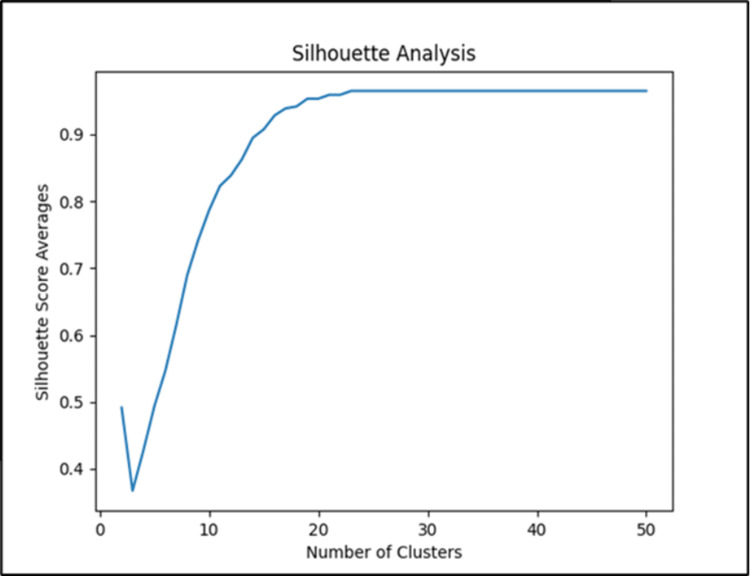
Silhouette Analysis of distal femur fracture data to determine the number of clusters.

Based on Silhouette Analysis (Figure 2), the number of clusters that produced unique and the most homogeneous groups in terms of the clinical characteristics of fracture fixation type and fracture classification was 15 clusters. With 15 clusters, we attained a Silhouette Score of 0.9078 which suggests a robust clustering structure [13]. Twelve of 15 clusters were 100% homogeneous, with all patients having the exact same fixation type and fracture classification. However, three of the 15 clusters were not homogeneous. These three clusters were homogeneous in fixation type and their most prominent fracture classifications only comprised approximately 60% of the patients within that cluster. Within Cluster K, 60.00% of patients had a fracture classification of 33A3.3, 20.00% had a fracture classification of 33A3.1, and another 20.00% had a fracture classification of 33B. Within Cluster I, 62.50% of patients had a fracture classification of 33A3.2, while 25.00% had a fracture classification of 33A3.1 and 12.50% had a fracture classification of 33A2.3. Within Cluster H, 66.67% of patients had a fracture classification of 33A2.3, while 16.67% had a fracture classification of 33A2.1 with the remaining 16.67% having a fracture classification of 33A3.3 (Table 10).

**Table 10:** Percentages of successful surgeries for each cluster, corresponding to the clinical characteristics of fracture classification and implant. Percentage of successful surgeries for each cluster, corresponding to the clinical characteristics of fracture classification and implant. The number of patients in each cluster is given parenthetically. The cluster name and number of patients are given in parentheses.

	Implant Type		
AO Fracture Classification	Plate	Nail	Nail and Plate
33A2.1	Cluster A (4)	Cluster B (12)	-
33A2.2	Cluster C (18)	Cluster D (11)	Cluster E (3)
33A2.3	Cluster F (14)	Cluster G (2)	Cluster H* (6)
33A3.1	-	-	-
33A3.2	Cluster I* (8)	-	-
33A3.3	Cluster J (11)	Cluster K* (5)	-
33B	Cluster L (17)	-	-
33C	Cluster M (44)	Cluster N (9)	Cluster O (5)

In terms of clinical outcomes, we found that extraarticular simple spiral fractures treated with a PL resulted in a success in 75.00% of patients within that cluster compared with a 100.00% success rate with those treated with an NL. An oblique simple fracture at the distal femoral metaphysis stabilized with an NP had a success rate of 66.67% compared with 72.73% in those treated with an NL (and 72.22% treated with a PL. Extraarticular transverse simple fractures demonstrated success in 50.00% of patients treated with an NL, 78.57% in patients treated with a PL, and 100.00% success in those treated with NP. Extraarticular fragmentary wedge fractures treated with a PL showed success in 75.00% of patients. Extraarticular multi-fragmentary wedge fractures (33A3.3) treated with a PL demonstrated success in 45.45% of patients compared to 40.00% in NL. Patients with a partial articular fracture who were treated with a PL had success in 88.35% of patients. Those with a complete intraarticular fracture treated with NP had success in 100.00% of cases compared with 61.63% success with PL and 77.78% with NL. All combinations of fracture fixation and fracture type not mentioned here did not have a corresponding cluster with those characteristics (Table 10).

## Discussion

Various fixation constructs are available for the management of distal femur fractures. However, there is not a clear consensus regarding optimal fixation methods for the treatment of these injuries, especially the more complex fracture patterns. This retrospective study not only explored clinical and radiographic outcomes of distal femur fractures (based on fracture classification) managed with either an NL, PL, or NP dual construct but also, through a pilot cluster analysis, uniquely implements a preliminary outcomes-based surgical decision-making tool with the potential to assist surgeons with implant selection in the future.

Fracture classification was found to be significantly associated with fixation type (p = 0.022) (Table 3). 33A fractures had the most variability with the type of fixation utilized, PL fixation being the most common (59%). Notably, 94% of 33B and 76% of 33C type fractures were treated with PL alone compared to NL fixation. This is perhaps due to these surgeons' preference for the lower rates of reoperation and complication with PL compared to NL, despite the fact that NL offers an improved return to function [15].

In a recent randomized controlled trial comparing retrograde NL with lateral locking PL, Dunbar et al. demonstrated no significant functional difference at one year between the two treatment methods and proposed that surgeons should choose the fixation strategy with which they are most comfortable [15]. However, this study included only metaphyseal distal femur fractures with or without simple intraarticular fractures and did not include more complex intraarticular fracture patterns with comminution that may be difficult to treat with NL fixation and largely dependent on surgeon expertise. For example, Nino et al. demonstrated favorable outcomes in treating these more complex fracture patterns by utilizing partially threaded 6.5 mm screws to stabilize the intercondylar fracture, followed by intramedullary nailing and use of 4.0 mm partially threaded screws to address additional fracture fragments [5].

Several studies combine all extraarticular distal femur fractures together when evaluating outcomes with regard to the fixation method [16-18]. However, depending on the specific extraarticular fracture pattern, one method of fixation may not always be superior. For example, our study demonstrates that extraarticular simple spiral fractures (33A2.1) treated with a PL resulted in success in 75.00% of patients within that cluster (n = 4) compared with a 100.00% success rate with those treated with an NL (n = 12). These similar outcomes are corroborated in the literature by two different cross-sectional observation studies by Ocalan et al. [19] and Howard et al. [20]. Wilson et al. also noticed these similar outcomes and corroborated the preference for plates in treating intra-articular fractures [21]. The patient who had an unsuccessful surgery after being treated with a PL was due to a 2-point drop in ambulatory status. A similar success rate was noted for 33A2.2 fractures treated with PL, NL, or NP. 33A3.3 fractures treated with a PL demonstrated success in 45.45% of patients (n = 11) compared to 40.00% in NL (n = 5) which suggests that a single construct may not always be sufficient and additional fixation may be needed for more comminuted extraarticular fracture patterns.

Our study also demonstrated a high success rate (13/14 or 92.9%) of distal femur fractures managed with an NP dual construct which is consistent with other studies [10,17]. The single patient who was deemed to not meet the criteria of a successful outcome was due to a 2-point drop in ambulatory status rather than other complications such as infection, non-union, or implant failure. This decrease in ambulatory status could be due to deconditioning as even a patient with a healed fracture and strong fixation construct may have difficulty with ambulation if not appropriately undergoing postoperative rehabilitation exercises.

In a retrospective study, Passias et al. found that 100% of patients (n = 8) treated with NP dual construct went on to fracture union but did have a higher procedure and total fluoroscopic time than treatment with an NL or a PL alone [10]. In a retrospective cohort study by Roszman et al., NP dual constructs led to decreased rates of reoperation compared with PL [22]. A systematic review by Kontakis et al. demonstrated existing evidence that NP constructs lead to earlier weight-bearing by combining the load-bearing properties of each implant [11]. In a retrospective case-control study of 67 femurs, Garala et al. found that the advantage of NP in providing earlier weight bearing was especially beneficial in patients 50 years or older [23]. Additional studies with larger sample sizes are needed to corroborate the findings in this study and to also evaluate infection rates given the increased operative time.

Limitations

Despite the incorporation of 10 years of retrospective chart review, our final sample size was relatively small due to patients being excluded for polytraumatic injuries that would impact postoperative recovery and confound our results. Consequently, there was a significant limitation in the cluster analysis. Three of the 15 clusters had one prominent fracture classification comprising approximately 60-70% of the patients within that cluster. With a larger sample size, we would expect to see greater homogeneity. Equally, the clustering algorithm consisted of only two variables (fixation type and fracture classification), as the relatively small sample size was not sufficient for the addition of other variables. Our inclusion of patients as young as 18 years old, although providing a greater breadth of possible distal femur fractures, may not be representative of the cases of distal femur fractures which routinely present to orthopaedic trauma centers (i.e., geriatric distal femur fractures). The inclusion of additional variables in the future would allow for greater granularity without additional loss of homogeneity which we would otherwise see in a small sample. In addition, determining radiographic union can be subjective and was largely based on the attending physician progress notes and review of X rays. An objective radiographic union score, such as the Modified RUST score, should be utilized in future studies [24]. Future studies would also necessitate the capture of more detailed information such as the presentation of infection, for those whose implants became infected. We would also consider whether additional surgeries were performed for any of the specific reasons for non-successful surgery; for example, whether or not additional surgeries were performed to treat infection. Sub-analysis regarding reasons behind changes in ambulatory status would also be warranted to determine whether factors related to the surgery or any comorbidities influenced decrease in ambulatory status. Therefore, future studies will invariably contain not only large sample sizes but more detailed analyses.

Considering the above-stated factors, definite clinical conclusions cannot be drawn from our current cluster analysis which serves only as a preliminary pilot study. Further studies with larger cohorts and inclusion of additional patient-specific characteristics such as BMI, CCI, smoking status, baseline corticosteroid use, diabetes, and the presence of osteopenia/osteoporosis, which all could impact clinical outcomes should be utilized. Through this approach, it may be possible to predict the rate of successful outcomes of patients with distal femur fractures based on the type of fixation method, fracture classification, and patient-specific characteristics in future machine learning models. This may optimize postoperative outcomes by providing an adjuvant tool for the orthopaedic surgeon to decide which fixation method is best for a specific patient, with less reliance on personal treatment preferences.

## Conclusions

Management of distal femur fractures can be complex as there are various fixation methods available and it is not always certain which specific implant is most optimal. This 10-year retrospective chart review demonstrates that 33A, 33B, and 33C fractures were all more likely to be treated with PL rather than NL or NP fixation. It also highlights the importance of considering subtypes of extraarticular distal femur fractures rather than grouping them all similarly because variable outcomes were seen based on the fixation method and fracture subtype. In addition, our results show promising outcomes with the use of NP dual constructs for certain 33A and 33C fractures which is consistent with other studies. Finally, with larger cohorts and the incorporation of patient-specific characteristics into cluster analysis and machine learning models, a valuable clinical tool could be developed to help guide orthopaedic surgeons in managing distal femur fractures.
